# High morbidity and mortality in children with untreated congenital deficiency of leptin or its receptor

**DOI:** 10.1016/j.xcrm.2023.101187

**Published:** 2023-09-01

**Authors:** Sadia Saeed, Roohia Khanam, Qasim M. Janjua, Jaida Manzoor, Lijiao Ning, Sharoon Hanook, Mickaël Canouil, Muhammad Ali, Hina Ayesha, Waqas I. Khan, I. Sadaf Farooqi, Giles S.H. Yeo, Stephen O'Rahilly, Amélie Bonnefond, Taeed A. Butt, Muhammad Arslan, Philippe Froguel

**Affiliations:** 1 Department of Metabolism, Digestion and Reproduction, Imperial College London, London, UK; 2 INSERM UMR 1283, CNRS UMR 8199, European Genomic Institute for Diabetes (EGID), Institut Pasteur de Lille, Lille, France; 3 University of Lille, Lille University Hospital, Lille, France; 4 KAM School of Life Sciences, Forman Christian College, Lahore, Pakistan; 5 Department of Physiology and Biophysics, College of Medicine and Health Sciences, National University of Science and Technology, Sohar, Oman; 6 Department of Paediatric Endocrinology, Children’s Hospital, Lahore, Pakistan; 7 Department of Statistics, Forman Christian College, Lahore, Pakistan; 8 Paediatric Endocrinology, Mayo Hospital, Lahore, Pakistan; 9 Department of Paediatrics, Punjab Medical College, Faisalabad, Pakistan; 10 The Children Hospital and the Institute of Child Health, Multan, Pakistan; 11 Medical Research Council Metabolic Diseases Unit, Wellcome-MRC Institute of Metabolic Science - Metabolic Research Laboratories, University of Cambridge, Cambridge, UK; 12 Department of Pediatrics, Fatima Memorial Hospital, Lahore, Pakistan

**Keywords:** monogenic obesity, leptin-signaling deficiency, age-related changes, mortality, morbidity, body growth, metabolism, oxidative stress, consanguinity

## Abstract

The long-term clinical outcomes of severe obesity due to leptin signaling deficiency are unknown. We carry out a retrospective cross-sectional investigation of a large cohort of children with leptin (LEP), LEP receptor (LEPR), or melanocortin 4 receptor (MC4R) deficiency (n = 145) to evaluate the progression of the disease. The affected individuals undergo physical, clinical, and metabolic evaluations. We report a very high mortality in children with LEP (26%) or LEPR deficiency (9%), mainly due to severe pulmonary and gastrointestinal infections. In addition, 40% of surviving children with LEP or LEPR deficiency experience life-threatening episodes of lung or gastrointestinal infections. Although precision drugs are currently available for LEP and LEPR deficiencies, as yet, they are not accessible in Pakistan. An appreciation of the severe impact of LEP or LEPR deficiency on morbidity and early mortality, educational attainment, and the attendant stigmatization should spur efforts to deliver the available life-saving drugs to these children as a matter of urgency.

## Highlights

• Remarkably high morbidity and mortality in children with LEP or LEPR deficiency
• High incidence of pulmonary and GI infections is main cause of deaths
• Oxidative stress levels notably higher in LEP than in LEPR or MC4R deficiency
• Emphasis on delivery of precision medication for affected children of this region

## Graphical abstract

**Figure:**
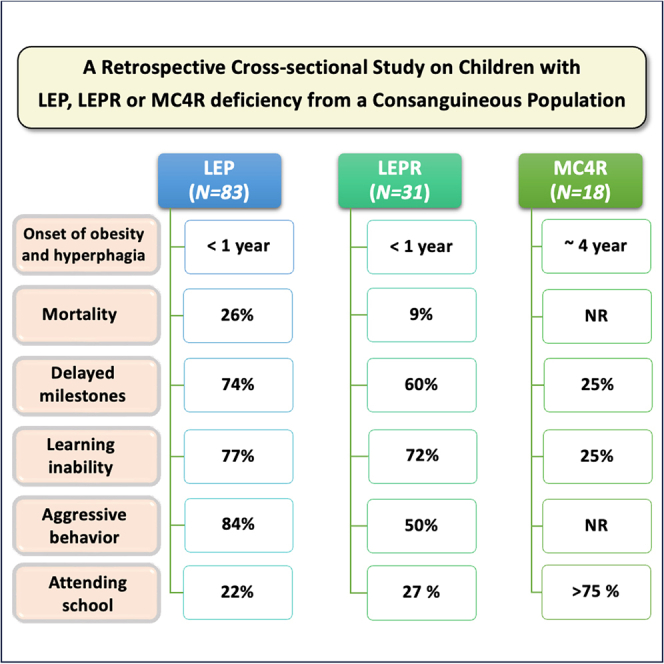

## Teaser

In this report, Saeed et al. investigate the natural history of leptin-signaling deficiency in a large cohort of affected children. The study reveals high morbidity and mortality in these patients at an early age. Lack of available treatment facilities for these patients calls for immediate action.

## Introduction

Biallelic, (likely) pathogenic variants in *LEP* encoding leptin and *LEPR* encoding its receptor, leptin receptor (LEPR), that follow highly penetrant Mendelian inheritance lead to a congenital LEP-signaling-deficient state, causing very-early-onset severe obesity due to persistent hyperphagia. The first evidence of the (likely) pathogenic variants in the *LEP* gene in the homozygous state resulting in LEP deficiency was reported in two obese cousins of Pakistani origin,^1^ whereas the first inactivating homozygous mutation in *LEPR* was found in three French sisters from Algeria.^2^ Since then, 22 pathogenic mutations in *LEP* and 55 in *LEPR* have been reported, most of them in highly consanguineous populations.^3^^,^^4^

The physiological role of LEP as a key initiator of hypothalamic neural circuitry that drives the central melanocortin pathway in the regulation of appetite is well established.^3^^,^^5^ LEP’s binding to its receptor results in the production of α- and β-melanocyte-stimulating hormones (α-MSH and β-MSH) from pro-opiomelanocortin (POMC), peptides that act as key satiety signals by activating the melanocortin 4 receptor (MC4R) on second-order neurons. Thus, LEP-driven central melanocortin signaling is the main central regulator of energy balance, food intake, and body weight.

Clinically, the initial descriptions of young patients with obesity consequent to LEP or LEPR deficiency were rather similar.^6^^,^^7^^,^^8^ Born with normal weight, the recessive mutation carriers gain excessive weight during infancy.^9^^,^^10^ In rodents and humans, LEP and LEPR deficiencies are associated with hyperinsulinemia, hypothyroidism, and hypogonadotropic hypogonadism resulting in delay or absence of pubertal development.^7^^,^^11^^,^^12^ Furthermore, there are some data to suggest that immune response of individuals with LEP-signaling deficiency may be seriously compromised with reduced numbers and proliferative function of T cells and vulnerability to infections during childhood.^13^^,^^14^^,^^15^ However, it is noteworthy that most of the existing clinical knowledge on LEP deficiency is based on individual case reports or on small pooled data from different ethnicities.^3^ Prospective studies of genetically homogeneous individuals with LEP-signaling deficiency have not been carried out so far. Thus, the long-term consequences of LEP deficiency are still unknown. Since 2012, we have constituted a cohort of children with severe obesity from consanguineous families for the study of genetic etiology of obesity in the Pakistani population (Severe Obesity in Pakistani Population [SOPP] study). We have previously reported an exceptionally high prevalence of monogenic obesity (49%) due to (likely) pathogenic variants in known obesity-associated genes, which included 33% of homozygous point mutations or copy-number variations (CNVs) in *LEP*, *LEPR*, and *MC4R*.^16^

Here, we present a retrospective clinical history of 92 children suffering from severe obesity due to LEP and 32 cases with LEPR deficiency, all from a single geographical region of Punjab, Pakistan. In addition, 21 cases of patients with severe obesity carrying (likely) pathogenic homozygous variants in *MC4R*, from the same population, have been included for comparison.

## Results

### Identification of mutations in SOPP cohort

The genetic screening of 454 families from our cohort, with severe obesity, identified 132 probands (29%) and 13 family members with homozygous or compound heterozygous (likely) pathogenic variants in *LEP*, *LEPR*, or *MC4R* (Table 1). Eighty-three children (53 males; 30 females) and nine family members (5 males; 4 females) carried 11 different homozygous (likely) pathogenic variants in *LEP.* These included five loss-of-function mutations (three frameshift and two splice site), one insertion or deletion (indel), and five missense mutations. With the exception of the two missense mutations, where cases presented high levels of circulating bioinactive LEP, the rest of the children presented with undetectable levels of LEP. The frameshift c.398del (p.G133Vfs∗15) was shown to be the most predominant *LEP* mutation identified in 62 patients (76%; 39 males and 23 females). This mutation was first reported in 1997 in a UK family of Pakistani origin.^1^ Interestingly, 54 probands carrying this mutation belonged to the Pakistani subethnicity Arain*,* suggesting a founder effect. For the remaining *LEP* mutations, no notable association with a particular region of origin or subethnicity was noticeable in this cohort (Table 1). Thirty-one probands (13 males; 18 females) and one family member were identified with 21 different (likely) pathogenic homozygous or compound heterozygous variants in *LEPR*. Among these, 17 were loss-of-function mutations including 5 splice site (n = 14), four nonsense (n = 5), one start loss (n = 1), three frameshift (n = 3), one compound heterozygous (n = 1), and three CNVs with homozygous deletions of various lengths (44–52 kb). The four missense (n = 4) mutations were identified in single cases, in SOPP cohort and were not identified in the other 1,328 children with severe obesity of the same ancestry recruited to the Genetics of Obesity Study (https://www.goos.org.uk) and, therefore, are likely to be private mutations. Eighteen probands (9 males; 9 females) and 3 family members (1 male; 2 females) were found to carry different homozygous mutations in *MC4R.* These included five missense, two nonsense, one frameshift, and one indel variants (Table 1).

**Table 1:** Pathogenic *LEP*, *LEPR*, and *MC4R* mutations identified in probands from SOPP cohort in relation to subethnic groups

	Mutation	Type of mutation	1^st^ reported/novel	REVEL score	No. probands	Subethnicity
*LEP*	c.398del (p.G133Valfs∗15)	frameshift	Montague et al.^1^	–	62	Arain (n = 54), Rajput, Mughal, Gazar, Jutt (n = 2), Tamboo, N/A^a^ (n = 2)
*LEP*	c.313C>T (p.R105W)	missense	Strobel et al.^17^	0.686 (Dam)	1	Sahotra
*LEP*	c.-28-16_-3del	splice site	Saeed et al.^8^	–	2	Mashki, Rajput
*LEP*	c.-29 + 1G>C	splice site	Saeed et al.^8^	–	4	Sheikh (n = 2), Sahotra, Lotay
*LEP*	c.298G>A (p.D100N)	missense	Saeed et al.^8^	0.610 (Dam)	1	Sheikh
*LEP*	c.309C>A (p.N103K)	missense	Mazen et al.^18^	0.523 (Dam)	2	Arain, Rehmani
*LEP*	c.314G>A (p.R105Q)	missense	Saeed et al.^16^	0.467 (Uncer)^b^	1	Ansari
*LEP*	c.350G>A (p.C117Y)	missense	Saeed et al.^8^	0.33 (Uncer)^b^	2	Warraich (n = 2)
*LEP*	c.104_106del (p.I35del)	in-frame deletion	Fatima et al.^19^	–	5	Rajput (n = 2), Jutt (n = 2), Bhatti
*LEP*	c.417delC (p.Y140Tfs∗8)	frameshift	Saeed et al.^16^	–	2	Syed Bukhari, Jutt
*LEP*	c.481_482del (p.L161Gfs∗10)	frameshift	Fatima et al.^19^	–	1	Jutt
*LEPR*	c.2396-1G>T	splice site	Saeed et al.^10^	–	7	Jutt, Rajput (n = 4), Ansari (n = 2)
*LEPR*	c.2396-2A>G	splice site	Saeed et al.^16^	–	2	Rajput, Bayi
*LEPR*	c.704-1G>A	splice site	Saeed et al.^16^	–	3	Ansari, Bhatti, Khansani
*LEPR*	c.2213-3C>G	splice site	Saeed et al.^16^	–	1	Kakazai
*LEPR*	c.994 + 2T>C	splice site	–	–	1	Mughal
*LEPR*	c.2T>C (p.?)	start loss	Saeed et al.^16^	–	1	Awan
*LEPR*	c.1674G>A (p.W558∗)	nonsense	Saeed et al.^10^	–	2	Siddiqui, Sheikh
*LEPR*	c.133_136dup p.(Tyr46∗)	nonsense	novel	–	1	Lalikher
*LEPR*	c.2114G>A (p.W705∗)	nonsense	Saeed et al.^16^	–	1	Dogar
*LEPR*	c.489T>A (p.Y163∗)	nonsense	novel	–	1	Barfizai
*LEPR*	c.1810T>A (p.C604S)	missense	Saeed et al.^16^	0.609 (Dam)	1	Gujar
*LEPR*	c.2627C>T (p.P876L)	missense	Saeed et al.^16^	0.647 (Dam)	1	Mughal
*LEPR*	c.40G>A (p.E14K)	missense	Saeed et al.^16^	0.116 (Ben)^c^	1	Rehmani
*LEPR*	c.2153A>G (p.N718S)	missense	Saeed et al.^16^	0.665 (Dam)	1	Jutt
*LEPR*	c.2899_2900insAT(p.A967Dfs∗7)	frameshift	Saeed et al.^16^	–	1	Jutt
*LEPR*	c.3268_3269del (p.S1090Wfs∗6)	frameshift	Bhatt et al.^20^	–	1	Arain
*LEPR*	c.1738delG(p.E580Kfs∗37)	frameshift	Saeed et al.^16^	–	1	Arain
*LEPR*	c.2875T>A (p.R959∗) and c.2872G>A (p.E958K)	compound heterozygous	Saeed et al.^16^	–	1	Gujar
*LEPR*	∼44 kb homozygous deletion including exons 3–20 of *LEPR*	CNV	Saeed et al.^16^	–	1	Gujar
*LEPR*	∼61 kb homozygous deletion including exons 3–6 of *LEPR*	CNV	Saeed et al.^8^	–	1	Rajput
*LEPR*	52 kb homozygous deletion	CNV	Saeed et al.^16^	–	1	Rajput
*MC4R*	c.633_636delCTAT (p.Y212Sfs∗5)	frameshift	Saeed et al.^16^	–	1	Khokhar
*MC4R*	c.47G>A (p.W16∗)	nonsense	Marti et al.^21^	–	3	Jutt, Rajput (n = 2)
*MC4R*	c.63_64del (p.Y21∗)	nonsense	Saeed et al.^16^	–	2	Arain (n = 2)
*MC4R*	c.601_612 del TTCTTCACCATG (p.F201_M204del)	in-frame deletion	Saeed et al.^16^	–	1	Rehmani
*MC4R*	c.206T>C (p.I69T)	missense	Tan et al.^22^	0.592 (Dam)	1	Jutt
*MC4R*	c.482 T>C (p.M161T)	missense	Tan et al.^22^	0.488 (Uncer)	3	Pathan (n = 2), Memon
*MC4R*	c.493C>T (p.R165W)	missense	Hinney et al.^23^	0.501 (Dam)	4	Arain (n = 2), Jutt Sayal, Lashari
*MC4R*	c.811T>C (p.C271R)	missense	Farooqi et al.^24^	0.844 (Dam)	1	Bhatti
*MC4R*	c.947 T>C (p.I316S)	missense	Yeo et al.^25^	0.442 (Uncer)	2	Rajput, Sheikh

LEP: NM_000230.3; LEPR: NM_002303.6; MC4R: NM_005912.3. Dam, damaging; Uncer, uncertain; Ben, benign.

a Not available.

b Undetectable levels of leptin hormone.

c Located at essential splice site.

### Clinical phenotypes

The most prominent and ubiquitous characteristic of children identified with biallelic (likely) pathogenic variants in *LEP*, *LEPR*, and *MC4R* genes was the onset of severe obesity and hyperphagia at a very young age—they lacked satiety and were constantly in demand of food. If denied, they demanded more food by crying and turning aggressive. The majority of the children with LEP or LEPR deficiency displayed excessive weight gain and hyperphagia that were noticed at a far earlier age in postnatal life (before 1 year of age; mean ages in children with LEP deficiency: 0.3 ± 0.04 years [n = 83] and in children with LEPR deficiency: 0.9 ± 0.1 years [n = 27]) as compared with those in children with MC4R deficiency (mean age: 4 ± 3.3 years [n = 20]).

**Figure 1:**
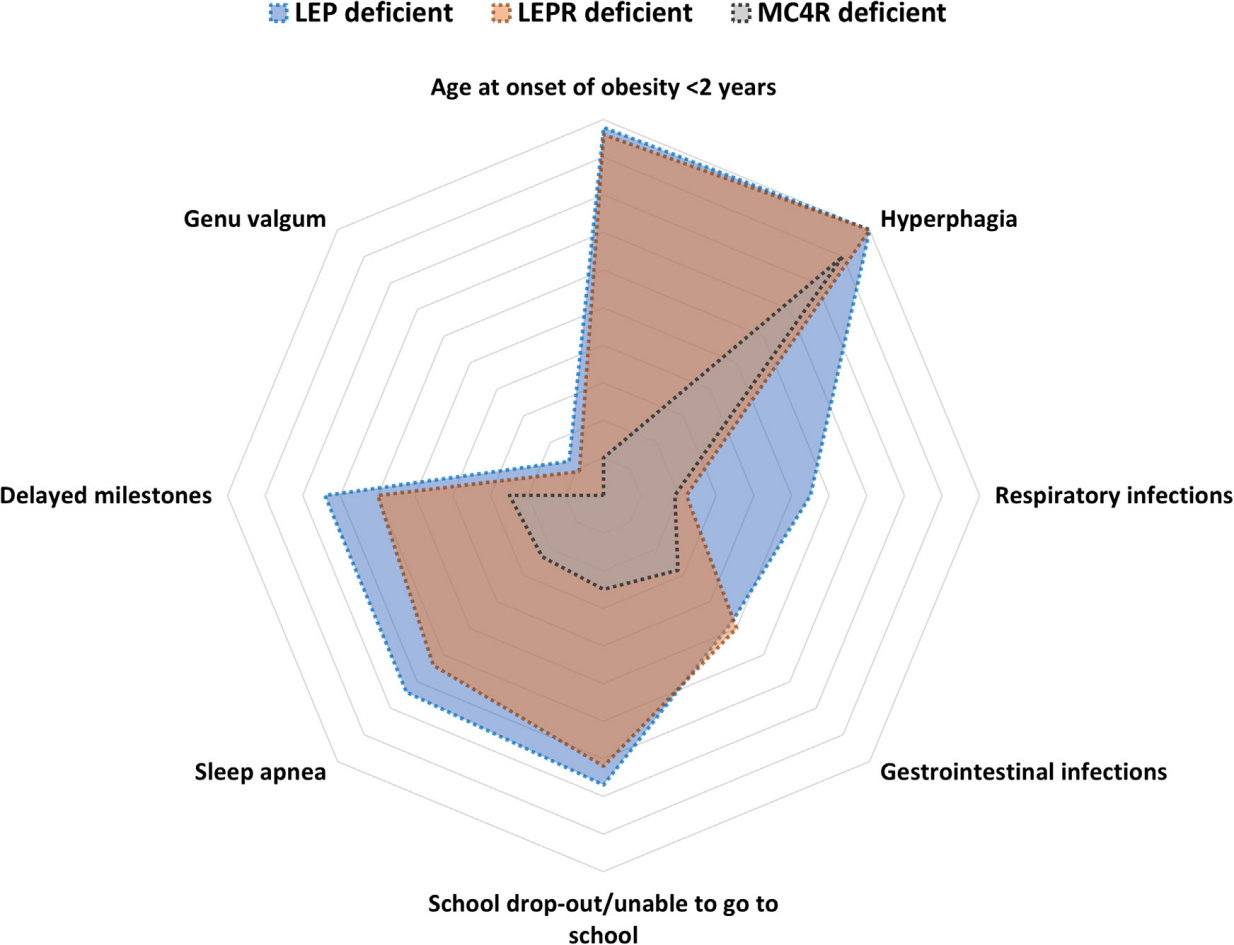
Radar chart representing the severity of the obesity-associated risks in children with LEP or LEPR deficiency compared with those with MC4R deficiency

Children deficient for LEP or LEPR presented a more precocious and profound hyperphagia than those with MC4R deficiency, with frequent aggressive demands for food during the day and when waking up at night. The craving for food was reported relatively less intense and frequent in children with MC4R deficiency. At the time of clinical examination, 84% of children with LEP, 79% with LEPR, and 61% with MC4R deficiency suffered from severe hyperphagia, whereas in the remaining affected subjects, the craving for food, though frequent, was reported as less severe or as moderate (Figure 1). It is noteworthy that with advancing age, no exacerbation of severity of hyperphagia was observed. On the contrary, a considerable variation in demand for food with time was observed in children with LEP or LEPR deficiency. This could partly be due to parents’ care and efforts to divert the attention of the child’s reward system to other healthy activities of choice.

No dysmorphic features or congenital malformations were observed in any of the obese probands. However, acanthosis nigricans was often present in the three mutant groups and was most predominant as dark velvety patches of skin around the neck (Figure S1A). Furthermore, prominent skin folds bracing the subcutaneous fat were invariably present on the forearms and trunk regions of children deficient for LEP or LEPR at an early stage of life but were less conspicuous in MC4R-deficient age-matched individuals (Figure S1B).

#### Morbidity

The majority of the children deficient for LEP or LEPR (55% and 38%, respectively) suffered from recurrent episodes of serious respiratory afflictions such as pneumonia and upper respiratory tract infections. Also, the majority of the children deficient for LEP or LEPR (74% and 64%, respectively) were assessed as hypoxic. The second most common complication observed is related to gastrointestinal infections often leading to severe diarrhea. About 40% of the living children with LEP or LEPR deficiency had one or more hospital admissions for intensive care management of these complications. In addition, genu valgum was identified in 13% of children deficient for LEP and 9% of children with LEPR deficiency but was not reported in the group with MC4R deficiency (Figure 1). Vision refractive errors were reported in 16% of probands with LEP deficiency but not in the other two groups.

#### Mortality

**Figure 2:**
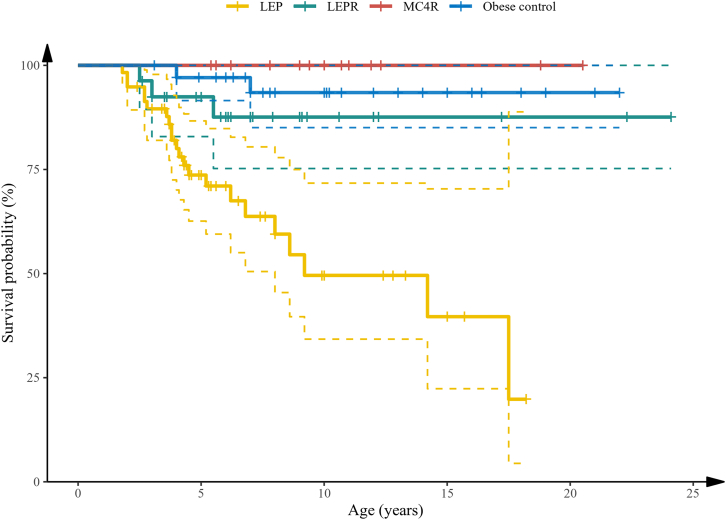
Survival curve of children with LEP, LEPR, and MC4R deficiencies and severely obese children negative for mutations in known obesity genes (obese controls) Kaplan-Meier group log-rank p < 0.05.

Deaths were reported in 26% of children with LEP deficiency and 9% of children with LEPR deficiency (Figure 1; Table S1). In the majority of these cases, the cause of death was diagnosed as respiratory failure due to pneumonia or other respiratory infections, and the second most common cause of death was severe diarrheal episodes. No deaths have so far been reported in children deficient for MC4R from this cohort. The Kaplan-Meier survival estimates demonstrate a remarkably low survival rate in children with LEP-signaling deficiency (p < 0.05; Figure 2).

### Physical activity and social behavior

Gross motor development milestones were noticeably delayed in 74% of children with LEP, 60% with LEPR, and 25% with MC4R deficiencies, presumably and partly due to excessive adiposity. Thus, the ability to independently sit and walk was acquired by these children at a much later stage compared with their non-obese siblings or with age-matched healthy subjects. Aggressive behavior in the majority of children with LEP or LEPR deficiency (84% and 50%, respectively) was reported toward their siblings and peers but not toward their parents. The relative inability to learn and remember new things was variable in affected children but was more evident in children with LEP or LEPR deficiency (77% and 72%, respectively) as compared with those of children with MC4R deficiency (25%). Overall, only a small percentage of the children with LEP deficiency (22%) or with LEPR deficiency (27%) over the age of 5 years were reported to be attending school compared with 75% of children with MC4R deficiency. Furthermore, those children who initially attended school very often dropped out prematurely mainly due to their inability to keep in step with their peers in studies, a lack of integration and acceptance by their peers, frequent health problems, and socio-psychological pressures (Figure 1). A lag in learning abilities as evidenced by difficulty in learning/remembering new things, delayed milestones, aggressive behavior, and high dropout rates from school in children with *LEP* and *LEPR* mutations probably indicate an age-related delay in cognitive ability. However, no attempt was made to quantitatively assess cognitive limitations in these patients.

#### Body growth

Longitudinal growth data in relation to age in the three mutant groups and in control subjects with healthy body weight from age 1–15 years are presented in Figure S2 and S2 and Tables S3A. The mean height was significantly greater (p < 0.05) in children with MC4R deficiency than in the other two study groups, whereas no significant differences in growth were found between children deficient for LEP or LEPR (Table S3A). The estimated linear growth in children with MC4R deficiency was also significantly increased compared with the age- and gender-matched controls (8.7 ± 2.3; p = 0.0001). We observed a similar trend (but statistically non-significant) in individuals with LEP or LEPR deficiency (1.7 ± 1.6 and 2.3 ± 1.9, respectively) (Table S4; Figure S2). No remarkable differences were found in longitudinal growth in individuals carrying mutations and the control subjects during the first 10 years of life (Table S3A). However, we noticed a trend toward an increased growth in individuals with MC4R deficiency in children ≥10 years old compared with age-matched children with LEP or LEPR deficiency and also compared with the control subjects. Age-related body weight and body mass data are summarized in Figure S3 and Tables S2, S3B, and S3C.

### Metabolic characteristics

The overall metabolic characteristics of children with mutations in the three genes and those with healthy body weight are presented in Table 2. As expected, insulin levels were significantly higher in the three mutant groups compared with control values and increased progressively with advancing age (Table S5A). At the age of 10–15 years, hyperinsulinemia appeared milder in the group with LEP deficiency as compared with the patients with LEPR and MC4R deficiencies (mean 49 versus 104 and 66 μIU/mL in children with LEP, LEPR, or MC4R deficiency, respectively) (range of insulin assay: 0–300 μIU/mL) (Table S5A).

**Table 2:** Endocrine and glycemic characteristics of children with *LEP*, *LEPR*, and *MC4R* mutations and lean controls

Characteristic	*LEP* deficient	*LEPR* deficient	*MC4R* deficient	Controls
Leptin, ng/mL (n)	0.1 ± 0.0^a^^,^^b^ (108)	31.9 ± 2.7^c^^,^^d^ (49)	26.7 ± 3.5^c^^,^^d^ (26)	3.1 ± 0.1^a^^,^^b^ (68)
Insulin, μIU/mL (n)	24.6 ± 2.3^a^^,^^b^^,^^d^ (111)	41.8 ± 5.9^c^^,^^d^ (49)	49.0 ± 10.3^c^^,^^d^ (26)	6.9 ± 0.5^a^^,^^b^^,^^c^ (61)
Cortisol, μg/dL (n)	16.2 ± 0.8^a^^,^^b^^,^^d^ (111)	13.3 ± 0.7^c^^,^^d^ (49)	10.9 ± 0.8^c^ (26)	9.4 ± 0.3^a^^,^^c^ (61)
TSH, μIU/mL (n)	2.2 ± 0.1 (112)	2.3 ± 0.2 (49)	2.1 ± 0.2 (26)	2.2 ± 0.1 (61)
HbA1C (n)	5.2 ± 0.1^d^ (29)	5.0 ± 0.1^b^^,^^d^ (19)	5.9 ± 0.5^a^^,^^d^ (7)	4.2 ± 0.1^a^^,^^b^^,^^c^ (40)
RBG∗, mg/dL (n)	95.7 ± 3.2 (30)	99.7 ± 3.7 (23)	109.1 ± 7.8 (15)	102.8 ± 2.6 (40)
T4, μg/dL (n)	8.4 ± 0.6 (23)	7.0 ± 0.5 (11)	7.6 ± 0.9 (15)	8.1 ± 0.3 (27)
T3, ng/mL (n)	1.8 ± 0.1 (23)	1.9 ± 0.2 (11)	1.6 ± 0.1 (15)	1.5 ± 0.1 (27)

Data represent mean ± SEM.

∗Random blood glucose

a Statistically significant p <0.05 (Scheffe’s multiple test) vs. LEPR deficient.

b Statistically significant p <0.05 (Scheffe’s multiple test) vs. MC4R deficient.

c Statistically significant p <0.05 (Scheffe’s multiple test) vs. LEP deficient.

d Statistically significant p <0.05 (Scheffe’s multiple test) vs. controls.

LEP concentrations in LEPR deficiency tended to be higher in the first 5 years of life than those of the children with MC4R deficiency (p < 0.05) (Table S5B). As anticipated, serum LEP concentrations were below the sensitivity level of the assay in children with LEP deficiency with the exception of two individuals (0.7 and 1.5 years of age) with the p.N103K mutation and one individual (2 years of age) with the p.D100N mutation in *LEP*. These cases present high levels of circulation of immunoreactive, but bioinactive, LEP protein (mean: 49 ng/mL) as also reported previously.^26^

Mean cortisol levels (morning) were significantly higher (p < 0.05) in LEP deficiency than those in the other two groups and tended to decline with age (Table S5C) but were indistinguishable from the lean controls in patients with MC4R deficiency (Table 2). Remarkably, mean serum thyroid-stimulating hormone (TSH), T4, and T3 concentrations were within the healthy range in children with LEP, LEPR, or MC4R deficiency and were not different from those of healthy subjects (Tables 2 and S5D). Mean HbA1c ranged between 5 and 5.9 in the three mutant groups compared to a mean of 4.2 in the control group (Table 2). Mean random blood glucose (RBG) levels were within the healthy range in the three mutant groups and were comparable with lean control values (Table 2), except in one individual with MC4R deficiency (RBG = 199 mg/dL).

### Oxidative stress

Peripheral levels of malondialdehyde (MDA) and 8-OHdG, critical biomarkers of oxidative stress that occurs as a consequence of an imbalance between the formation of free oxygen radicals and inactivation of these species by the antioxidant defense system, and of glutathione (GSH), an antioxidant biomarker, were measured in a restricted number of probands. Serum levels of MDA and 8-OHdG were significantly higher and of GSH lower in individuals with LEP or LEPR deficiency compared with those with MC4R deficiency (p < 0.05) (Table S5E). Circulating levels of MDA and 8-OHdG were maximal in children with LEP deficiency.

## Discussion

Our study demonstrates a high mortality rate in obese children with LEP deficiency and to a lesser extent in children with LEPR deficiency. While increased susceptibility to infection and mortality have been reported in case reports of these conditions previously,^7^^,^^27^ this large clinical series of children definitively establishes the severe clinical impact of these conditions. In contrast, no severely obese child with MC4R deficiency of same ethnic origin died during the study. Indeed, all children with severe obesity in this study belong to the same geographical region and share similar socioeconomic status (low middle economy). Previous studies indicate that the country’s disease burden may be overtly influenced by the socio-economic index.^28^ In LEP-signaling deficiency, the mortality rate may even be underestimated, as ∼30% of the families could not be contacted following the initial presentation. Health records indicated that deaths of the children were mostly due to respiratory and gastrointestinal infections. These effects may be due to lowered immunity and possibly a lack of a permissive effect of LEP on immune cells.^13^^,^^29^

Previously, clinical investigation of three patients with LEP deficiency demonstrated an increased frequency of infections predominantly of the respiratory tract when compared with their wild-type siblings. T cell number and function were impaired in the subjects with a reduced number of CD4^+^ T cells, reduced T cell proliferation, and cytokine response, but no gross abnormalities related to thermoregulation were reported.^13^ These findings of T cell responsiveness were consistent with observations in *ob/ob* mice.^30^ Therapeutic doses of recombinant LEP normalized the T cell function.^13^ These findings suggest that LEP is a crucial metabolic signal that allows activation and proliferation of T cells, thus linking nutritional status, cellular metabolism, and immunity.^31^

Another outcome of this investigation is the observation that as many as 40% of living young patients with LEP or LEPR deficiency had experienced serious health problems posing a life-threatening risk and necessitating hospitalization for intensive care management. According to a recent UNICEF (The United Nations Children’s Fund) report, mortality in children under 5 years of age (excluding neonatal mortality, which constitutes more than half of the under-5 deaths in the country) is estimated at 2.5% in Pakistan for the year 2020 (Monitoring the situation of children and women. Pakistan, Key demographic indicators 2020: https://data.unicef.org/country/pak/). Our data unequivocally demonstrate that LEP deficiency leads to a remarkably higher mortality rate, presumably due to lowered immunity leading to high risk of infections in these children. Also, according to the Global Burden of Diseases, Injuries, and Risk Factors Study (GBD) 2019, neonatal disorders, congenital defects, diarrheal diseases, and respiratory infections have been shown to be among the top ten main causes of premature mortality and years of life lost in Pakistan.^28^

Although severe obesity has been considered a long-term risk factor of early morbidity, disability, and premature death in adulthood, here we present a well-defined non-syndromic form of genetic obesity in childhood shown to be so dramatically life threatening. Indeed, more common forms of severe early-onset obesity are associated with metabolic and cardiovascular disease, as well as with some cancers later in life, but not with high infection rates (with the recent exception of COVID19 in relatively young adults). Earlier research on LEP-signaling deficiency in human and animals suggested impaired immunity that may contribute to the severity of common respiratory and digestive infections.^13^ Although we were not able to carry out direct evaluation of humoral and cellular immunity in our children with obesity, we found a high systemic oxidative stress level and a marked depletion of the antioxidant GSH in obese carriers with homozygous loss-of-function mutations in *LEP* or *LEPR* genes. In comparison, the oxidative stress level was found to be lower in patients with MC4R deficiency. Earlier studies suggested a positive correlation between body mass index and levels of oxidative stress,^32^^,^^33^ but here we demonstrate that the level of oxidative stress related to obesity may differ even among well-defined conditions of genetic obesity. In spite of a close phenotypic identity between *LEP* and *LEPR* mutation carriers, morbidity in children with LEP deficiency is surprisingly much higher than that of patients with LEPR deficiency. We also found a higher index of oxidative stress and DNA damage in children deficient for LEP compared with those with LEPR deficiency, suggesting a more severe impairment in cellular or humoral immunity of unknown nature.

The very early onset of weight gain due to severe hyperphagia in children with LEP or LEPR deficiency is well documented,^6^ and in this respect, we confirm that children with LEP or LEPR deficiency are indistinguishable.^7^^,^^16^^,^^29^ In addition, our ethnically homogeneous study unambiguously shows that the onset of severe obesity is ∼3 years earlier in both LEP and LEPR deficiencies compared with the children deficient for MC4R.

Here, we also document the high incidence of delay in learning ability, possibly due to intellectual impairment, and aggressive behavior in children with LEP or LEPR deficiency that appear to be milder or absent in children with MC4R deficiency. This is also evidenced by the observation that only ∼25% of children with LEP or LEPR deficiency could attend school after the age of 5 years compared with 75% of children with MC4R deficiency. Also, long-term schooling was nearly impossible for children deficient for LEP or LEPR. This other dramatic outcome is further supported by our recent study on a group of severely obese teenagers from the same geographical region and with the same level of consanguinity (mean BMI = 37 kg/m^2^; mean age = 18 years) who were regularly attending school. Remarkably, this cohort did not include a single subject carrying a mutation in the *LEP* gene.^34^

LEP-deficient *ob/ob* and LEPR-deficient *db/db* mice have decreased linear body growth.^35^ In human, previous reports on linear growth due to defective LEP or LEPR signaling have been controversial,^2^ with reports of both accelerated^36^^,^^37^ and diminished^2^^,^^38^ linear growth. Here, our data in a much larger cohort demonstrate no remarkable differences in linear growth in children with LEP or LEPR deficiency compared with age-matched lean controls up to the age of 15 years. In the present investigation, we observed a significantly increased mean linear growth in children with MC4R deficiency as compared with the other two groups of mutation carriers. Previously, MC4R deficiency has also been associated with increased linear growth due to enhanced osteogenesis.^24^

Hyperinsulinemia, though significantly more pronounced in individuals with LEPR or MC4R deficiency compared with those with LEP deficiency, increased with age in all three mutant groups. Raised HbA1c levels (but still within the healthy range) were found in children with MC4R deficiency but to a lesser extent in children deficient for LEP or LEPR. Consequently, none of these children had thus far developed a risk of diabetes. The euglycemic condition in affected children in this study is in concordance with previous observations.^24^

Hypercortisolemia was more prominent in children with LEP deficiency compared with that in patients with LEPR deficiency, whereas cortisol levels were in the healthy range in patients with MC4R deficiency and were indistinguishable from those of the lean controls, as previously reported.^8^ This corresponds to the high cortisol levels seen in *ob*/*ob* and *db*/*db* mice.^39^^,^^40^ Observations on thyroid function in subjects with these mutations were again conflicting.^13^^,^^41^ Notably, in this large study, serum TSH, T4, and T3 levels were within the healthy range in all the three mutant groups, thus excluding thyroid abnormalities in children in this study. However, here no attempt was made to estimate free T4 levels.

### Conclusions

In summary, comparative data from this retrospective cross-sectional study indicate a distinctly higher level of morbidity in children with LEP or LEPR deficiency compared with those with homozygous loss-of-function mutations in the *MC4R* gene. Current or novel medications against monogenic forms of obesity, though available in many developed countries, are unfortunately lacking in Pakistan—a country with the world’s highest recorded prevalence of LEP-signaling deficiency. The treatments include hormone replacement therapy with recombinant leptin for subjects with LEP deficiency, the MC4R agonist setmelanotide for LEPR deficiency,^42^ and glucagon-like peptide-1 receptor agonists for subjects with MC4R deficiency.^43^^,^^44^ The fact that a sizable population of children are failing to achieve normal educational development and are becoming seriously ill and dying prematurely as the result of a deficiency in hormonal signaling for which relatively simple peptide treatments are readily available highlights serious flaws in the global system through which drugs are developed and made available to those who most need them.

### Limitations of the study

This investigation has certain limitations. It is necessarily a cross-sectional study and lacks the advantage of an organized follow-up regimen. A regular follow-up was not possible because of logistic problems, as a sizable proportion of affected families, especially those residing in rural and remote areas of the province/country, did not respond to a follow-up call or could not be contacted the second time.

## STAR★Methods

### Key resources table

**Table:** 

REAGENT or RESOURCE	SOURCE	IDENTIFIER
**Critical commercial assays**		

Leptin	Monobind	Cat# 10825-300A
Cortisol	Monobind	Cat# 3625-300A
Insulin	Monobind	Cat# 2425-300A
Thyroid-stimulating hormone (TSH)	Monobind	Cat# 6025-300A
8-Hydroxy-2′-deoxyguanosine (8-OHdG)	Elabsciences	Cat# E-EL-0028
Glucose	Certeza Medical	Cat# GL-110
HbA1c	Biohermes HbA1c	Cat# HbA1c EZ 2.0

**Software and algorithms**		

SPSS 25	IBM	https://www.ibm.com/products/spss-statistics
R 4.0.2	The R Foundation	https://cran.r-project.org/bin/windows/base/

### Resource availability

#### Lead contact

Further information and requests for resources and reagents should be directed to the lead contact, Dr. Sadia Saeed (s.saeed08@imperial.ac.uk)

#### Materials availability

This study did not generate new unique reagents.

### Experimental model and study participant details

#### Human subjects

This retrospective cohort study is based on 145 cases (132 probands and 13 siblings) of monogenic obesity due to homozygous loss-of-function mutations in the *LEP, LEPR* and *MC4R* genes (Table S1). These mutations were identified through genetic screening of 454 unrelated children with severe early-onset obesity recruited to the SOPP [Severe obesity in Pakistani Population] study, from consanguineous population of Pakistan. The inclusion criteria were based on a body mass index standard deviation score (BMI SDS) of ≥3.5 and age of obesity onset ≤5 years. Where possible, other affected family members were also included in the study. In addition, a group of age-matched children negative for these mutations and of normal body weight have been included as lean controls.

#### Ethics statement

The study was approved by the relevant institutional ethical committees, and all participants or their parents/guardians provided written informed consent. The study was conducted according to the principles outlined in the Declaration of Helsinki. A detailed interview with the patient and/or guardian, was carried out and medical history was recorded at the time of first recruitment as well as at the time of any follow-up examination. Anthropomorphic measurements were carried out and blood samples obtained (between 10 a.m.–12 noon), for subsequent genetic and hormonal analysis.

### Method details

#### Genetic analysis

Initially, all the 454 probands with severe obesity were genetically screened for mutations in *LEP* and *MC4R* genes through Sanger sequencing. DNA of the probands found negative for the mutations in these two genes, was further analyzed through conventional or augmented whole exome sequencing (WES). The pathogenicity of the mutations was determined by following the American College of Medical Genetics and Genomics (ACMG) criteria. The screening methods have been described in detail elsewhere.^34^^,^^45^ Sixty-three of these affected children (56 probands and 7 siblings) were also clinically screened at a more advanced age but at variable intervals. Necessarily, we have included data obtained from these patients at their subsequent visit (or follow-up) as independent values in our age-related cross-sectional observations.

#### Biochemical determinations

Metabolic and oxidative stress biomarkers were measured in serum. Leptin, insulin, cortisol, thyroid-stimulating hormone (TSH), and 8-hydroxy-2′-deoxyguanosine (8-OHdG), were determined using commercially available ELISA kits (leptin, insulin, cortisol, TSH: Monobind, Lake Forest, USA; 8OHdG: Elabscience, Houston, USA). Serum levels of malondialdehyde (MDA) and glutathione (GSH) were determined spectrophotometrically. Samples were analyzed in duplicate. The intra- and inter-assay variations were less than 11% for each assay. Random blood glucose and glycated hemoglobin A1c (HbA1c) were determined at presentation.

### Quantification and statistical analyses

#### Statistical analysis

Comparisons between the traits were made using Scheffe’s test. For association analysis between trait and study groups, we applied a linear regression model on different traits to assess the effect of the *LEP, LEPR* and *MC4R* mutant groups compared to the lean control group adjusted by gender and age. p-values <0.05 were considered statistically significant. Since the number of children in the three mutant groups, was not equally distributed across age (or in relation to age), the present data have also been analyzed in three consecutive windows of 5 years for comparative purposes. The results were analyzed using the SPSS data analysis program.

The families of the three mutant groups as well as obese children with yet undiagnosed causes were approached for reporting any deaths among the affected individuals. Survival in the three mutant groups and obese controls was estimated in cases of death, and at the age of the last reported visit or contact by phone. Survival curves were estimated and plotted among the three mutant groups and obese controls, using the Kaplan-Meier method with R.

## Data Availability

All data reported in this paper will be shared by the lead contact upon request. This paper does not report original code. Any additional information required to reanalyse the data reported in this paper is available from the lead contact upon reasonable request.

## Supplemental information

Document S1. Figures S1–S3 and Tables S1–S5

Document S2. Article plus supplemental information
