# Supplementary material for: High morbidity and mortality in children with untreated congenital deficiency of leptin or its receptor
Source: Cell Rep Med. 2023 Sep 1;4(9):101187. doi: 10.1016/j.xcrm.2023.101187 (PMC10518629; doi:10.1016/j.xcrm.2023.101187)
Supplement: Document S1. Figures S1–S3 and Tables S1–S5 [file mmc1.pdf]

Cell Reports Medicine, Volume *4*

Supplemental information

High morbidity and mortality in children with untreated congenital deficiency of leptin or its receptor

Sadia Saeed, Roohia Khanam, Qasim M. Janjua, Jaida Manzoor, Lijiao Ning, Sharoon Hanook, Mickaël Canouil, Muhammad Ali, Hina Ayesha, Waqas I. Khan, I. Sadaf Farooqi, Giles S.H. Yeo, Stephen O'Rahilly, Amélie Bonnefond, Taeed A. Butt, Muhammad Arslan, and Philippe Froguel

Supplementary Information

Supplementary Tables

Table S1. Mortality in severely obese patients with LEP, LEPR and MC4R deficiencies and with unknown genetic cause, and in normal body weight controls. Related to Figures 1 and 2

Group	No. of families (n)	Reported mortality (n)	Mortality %	Age range (y)*
LEP deficient	83	22	26	1.8-17.5
LEPR deficient	31	3	9	2.5-5.5
MC4R deficient	18	0	0	NA**
Obesity with unknown genetic cause	35	2	5.7	4.0-7.0
Lean controls	68	0	0	NA**

* Refers to age at mortality
** Not applicable

Table S2. Physical characteristics of severely obese children with *LEP, LEPR* and *MC4R* mutations and normal controls. Related to Results: Body Growth

**Characteristic**	***LEP* deficient**	***LEPR* deficient**	***MC4R* deficient**	**Controls**
n	**112**	**49**	**27**	**69**
Age (years)	3.3 ± 0.3 $^{c,\,d}$	4.3 ± 0.6 ^{d}	6.0 ± 0.8 ^{a}	6.5 ± 0.4 $^{a,\,b}$
Height (cm)	92.2 ± 2.4 $^{c,\,d}$	99.1 ± 4.0 ^{c}	116.6 ± 5.2 $^{a,\,b}$	111.3 ± 2.8 ^{a}
Weight (Kg)	33.0 ± 2.3 $^{c,\,d}$	36.8 ± 3.7 $^{c,\,d}$	52.8 ± 6.2 $^{a,\,b,\,d}$	21.6 ± 1.6 $^{a,\,b,\,c}$
BMI SDS for age	7.9 ± 0.3 ^{d}	7.7 ± 0.5 ^{d}	6.5 ± 0.5 ^{d}	-0.3 ± 0.2 $^{a,\,b,\,c}$

Superscripts indicate statistically significant P < 0.05 (Scheffe's multiple test); ^{a} vs LEP deficient, ^{b} vs LEPR deficient, ^{c} vs MC4R deficient, ^{d} vs controls

Data represents Mean ± SEM

Table S3. Age-related changes in physical characteristics in children carrying *LEP, LEPR* and *MC4R* mutations and age-matched normal controls. Related to Results: Body Growth

Age group (y)	n	*LEP* Mean (SEM)	n	*LEPR* Mean (SEM)	n	*MC4R* Mean (SEM)	n	Control Mean (SEM)
**A-Longitudinal height** (cm)								
≤1-4.9	84	80.7 ± 1.5	30	80.0 ± 2.3	10	90.0 ± 4.5	22	87.9 ± 2.7
5-9.9	20	118.3 ± 2.7	14	125.3 ± 3.0 ^{d}	11	120.6 ± 3.5	31	112.5 ± 1.9 ^{b}
10-15	8	147.8 ± 2.9	5	140.6 ± 8.3	6	153.8 ± 4.5	16	141.1 ± 4.5
Cumulative	*112*	92.2 ± 2.4 $^{c, d}$	*49*	99.1 ± 4.0 ^{c}	*27*	116.6 ± 5.2 $^{a, b}$	*69*	111.3 ± 2.8 ^{a}
**B- Body weight** (kg)								
≤1-4.9	84	22.6 ± 1.1 ^{d}	30	21.2 ± 1.4 ^{d}	10	24.1 ± 3.0 ^{d}	22	11.8 ± 0.6 $^{a, b, c}$
5-9.9	20	52.9 ± 4.5 ^{d}	14	50.4 ± 3.8 ^{d}	11	51.8 ± 4.8 ^{d}	31	18.4 ± 0.6 $^{a, b, c}$
10-15	8	91.8 ± 5.4 ^{d}	5	92.5 ± 11.0 ^{d}	6	102.7 ± 4.9 ^{d}	16	41.2 ± 3.6 $^{a, b, c}$
Cumulative	*112*	33.0 ± 2.3 $^{c, d}$	*49*	36.8 ± 3.7 $^{c, d}$	*27*	52.8 ± 6.2 $^{a, b, d}$	*69*	21.6 ± 1.6 $^{a, b, c}$
**C- BMI SDS**								
≤1-4.9	84	8.2 ± 0.3 ^{d}	30	8.6 ± 0.7 ^{d}	10	7.0 ± 0.8 ^{d}	22	-0.3 ± 0.3 $^{a, b, c}$
5-9.9	20	8.1 ± 0.8 ^{d}	14	6.3 ± 0.8 ^{d}	11	7.0 ± 0.7 ^{d}	31	-0.7 ± 0.2 $^{a, b, c}$
10-15	8	4.7 ± 0.4 ^{d}	5	5.8 ± 0.3 ^{d}	6	4.9 ± 0.7 ^{d}	16	0.6 ± 0.4 $^{a, b, c}$
Cumulative	*112*	7.9 ± 0.3 ^{d}	*49*	7.7 ± 0.5 ^{d}	*27*	6.5 ± 0.5 ^{d}	*69*	-0.3 ± 0.2 $^{a, b, c}$

Superscript letters indicate statistically significant $P < 0.05$ (Scheffe's multiple test); ^{a} vs LEP deficient, ^{b} vs LEPR deficient, ^{c} vs MC4R deficient, ^{d} vs controls.

Data represent Mean ± SEM

Table S4. Statistical analysis of age-related changes in height in *LEP, LEPR,* and *MC4R* deficient children and normal body weight controls. Related to Results: Body Growth

Terms	Estimate	Std Error	Statistics	P. value
Age	6.5	0.2	37.7	2.311
Gender	1.1	1.3	0.9	0.396
LEP (group)	1.7	1.6	1.0	0.302
LEPR (group)	2.3	1.9	1.2	0.229
MC4R (group)	8.7	2.3	3.9	0.000
Intercept	68.4	1.8	38.0	3.869

Statistical model: Height ~ age + gender + group
Trait: Height (cm)

Table S5. Age-related changes in biochemical characteristics and stress biomarkers in children carrying *LEP, LEPR* and *MC4R* mutations and age-matched normal controls. Related to Results: Metabolic Characteristics

Age group (y)	n	*LEP* Mean (SEM)	n	*LEPR* Mean (SEM)	n	*MC4R* Mean (SEM)	n	Control Mean (SEM)
**A-Insulin** (µIU/ml)								
≤1-4.9	83	21.9 ± 2.3	30	31.1 ± 4.9 [d]	9	38.9 ± 13.2 [d]	19	7.8 ± 0.8 [b, c]
5-9.9	20	26.2 ± 5.7	14	42.3 ± 9.8 [d]	11	47.7 ± 11.6 [d]	30	6.9 ± 0.9 [b, c]
10-15	8	48.7 ± 15.3	5	104.2 ± 32.4 [d]	6	66.4 ± 36.1	12	5.3 ± 0.5 [b]
Cumulative	*111*	24.6 ± 2.3 [b, c, d]	*49*	41.8 ± 5.9 [a, d]	*26*	49.0 ± 10.3 [a, d]	*61*	6.9 ± 0.5 [a, b, c]
**B-Leptin** (ng/ml)								
≤1-4.9	80	0.1 ± 0.0 [b, c]	30	35.0 ± 3.6 [a, c, d]	9	19.9 ± 4.5 [a, b, d]	22	2.6 ± 0.1 [b, c]
5-9.9	20	0.1 ± 0.0 [b, c]	14	23.8 ± 2.7 [a, d]	11	24.9 ± 5.3 [a, d]	31	3.3 ± 0.2 [b, c]
10-15	8	0.0 ± 0.0 [b, c]	5	36.0 ± 12.9 [a, d]	6	40.5 ± 7.2 [a, d]	15	3.6 ± 0.3 [b, c]
Cumulative	*108*	0.1 ± 0.0 [b, c]	*49*	31.9 ± 2.7 [a, d]	*26*	26.7 ± 3.5 [a, d]	*68*	3.1 ± 0.1 [b, c]
**C- Cortisol** (µg/dl)								
≤1-4.9	83	17.2 ± 0.9 [c, d]	30	14.5 ± 0.9	9	10.5 ± 0.8 [a]	19	9.2 ± 0.7 [a]
5-9.9	20	13.6 ± 1.5	14	11.1 ± 1.1	11	10.4 ± 1.6	30	9.9 ± 0.4
10-15	8	12.9 ± 2.8	5	12.3 ± 0.7	6	12.6 ± 1.6	12	8.4 ± 0.5
Cumulative	*111*	16.2 ± 0.8 [b, c, d]	*49*	13.3 ± 0.7 [a, d]	*26*	10.9 ± 0.8 [a]	*61*	9.4 ± 0.3 [a, b]
**D- TSH** (ulU/ml)								
≤1-4.9	84	2.2 ± 0.1	30	2.1 ± 0.2	9	1.8 ± 0.4	19	1.8 ± 0.1
5-9.9	20	2.2 ± 0.3	14	2.9 ± 0.7	11	2.2 ± 0.3	30	2.3 ± 0.2
10-15	8	2.5 ± 0.3	5	1.7 ± 0.4	6	2.2 ± 0.7	12	2.3 ± 0.2

Cumulative	*112*	2.2 ± 0.1	*49*	2.3 ± 0.2	*26*	2.1 ± 0.2	*61*	2.2 ± 0.1

E- Oxidative stress markers

MDA (nmol/ml)	3.20 ± 0.24 $^{c, d}$	3.54 ± 0.14 $^{c, d}$	1.35 ± 0.05 $^{a, b, d}$	0.66 ± 0.05 $^{a, b, c}$
(n)	(11)	(10)	(8)	(16)
GSH (µg/dl)	1.33 ± 0.12 $^{c, d}$	1.40 ± 0.05 $^{c, d}$	2.98 ± 0.23 $^{a, b, d}$	4.82 ± 0.32 $^{a, b, c}$
(n)	(11)	(10)	(8)	(16)
8-OHdG (ng/ml)	25.8 ± 3.2 ^{c}	21.0 ± 2.5 ^{c}	9.2 ± 1.3 $^{a, b}$	4.3 ± 0.2 $^{a, b}$
(n)	(28)	(19)	(6)	(12)

Superscript letters indicate statistically significant $P < 0.05$ (Scheffe test); ^{a} vs LEP deficient, ^{b} vs LEPR deficient, ^{c} vs MC4R deficient, ^{d} vs controls.
Data represent Mean ± SEM

Supplementary Figures

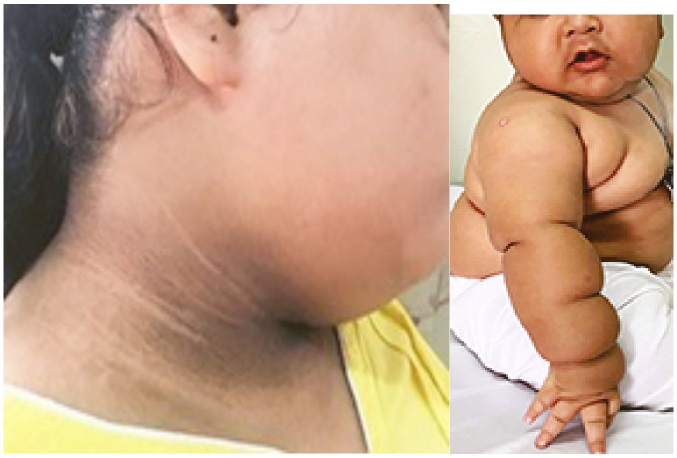

(a) **(b)**

Supplementary Figure 1. (a) Acanthosis nigricans and (b) prominent sub-cutaneous adipose tissue in two *LEP* deficient children. Related to results: Clinical Phenotype.

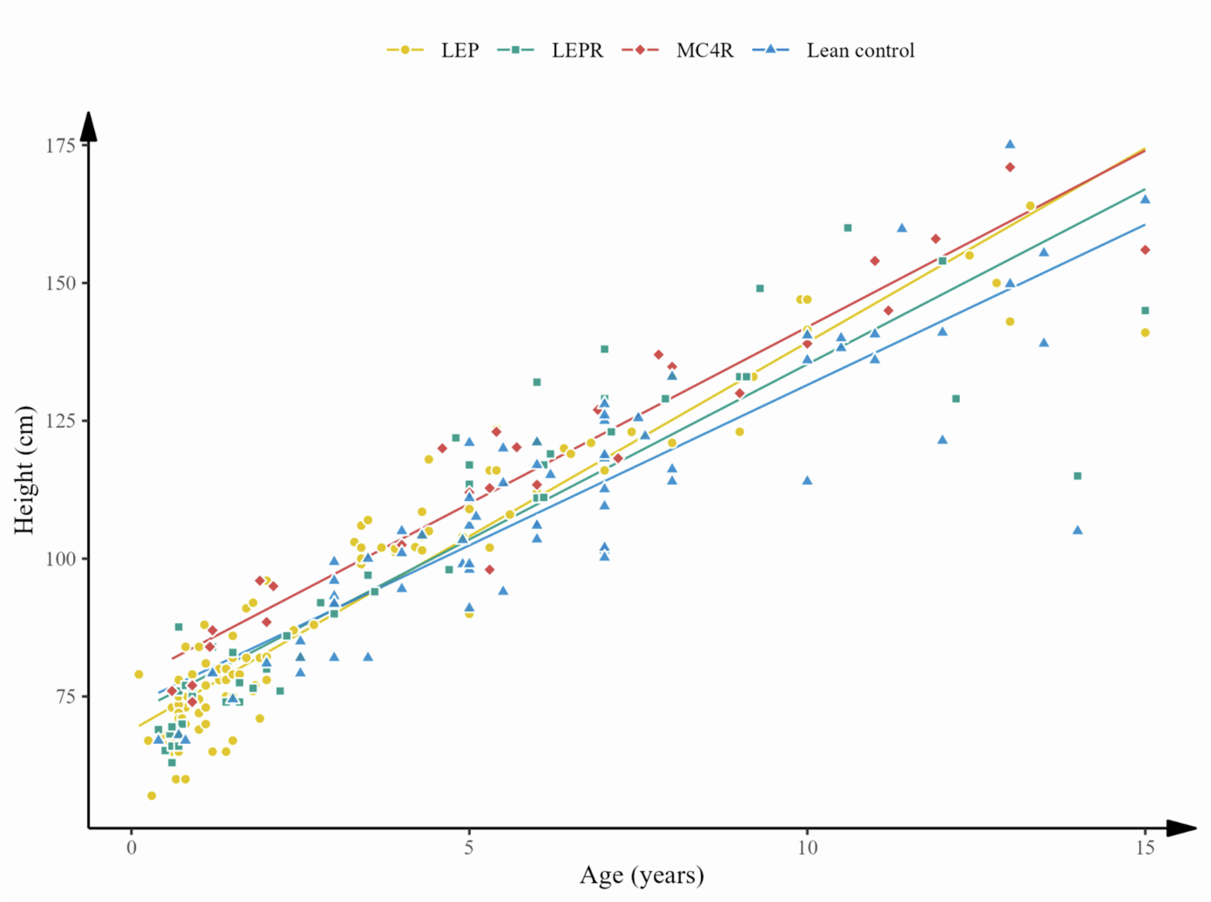

Supplementary Figure 2. Age-related changes in height in *LEP, LEPR* and *MC4R* deficient children and normal body weight controls. Related to Results: Body Growth.

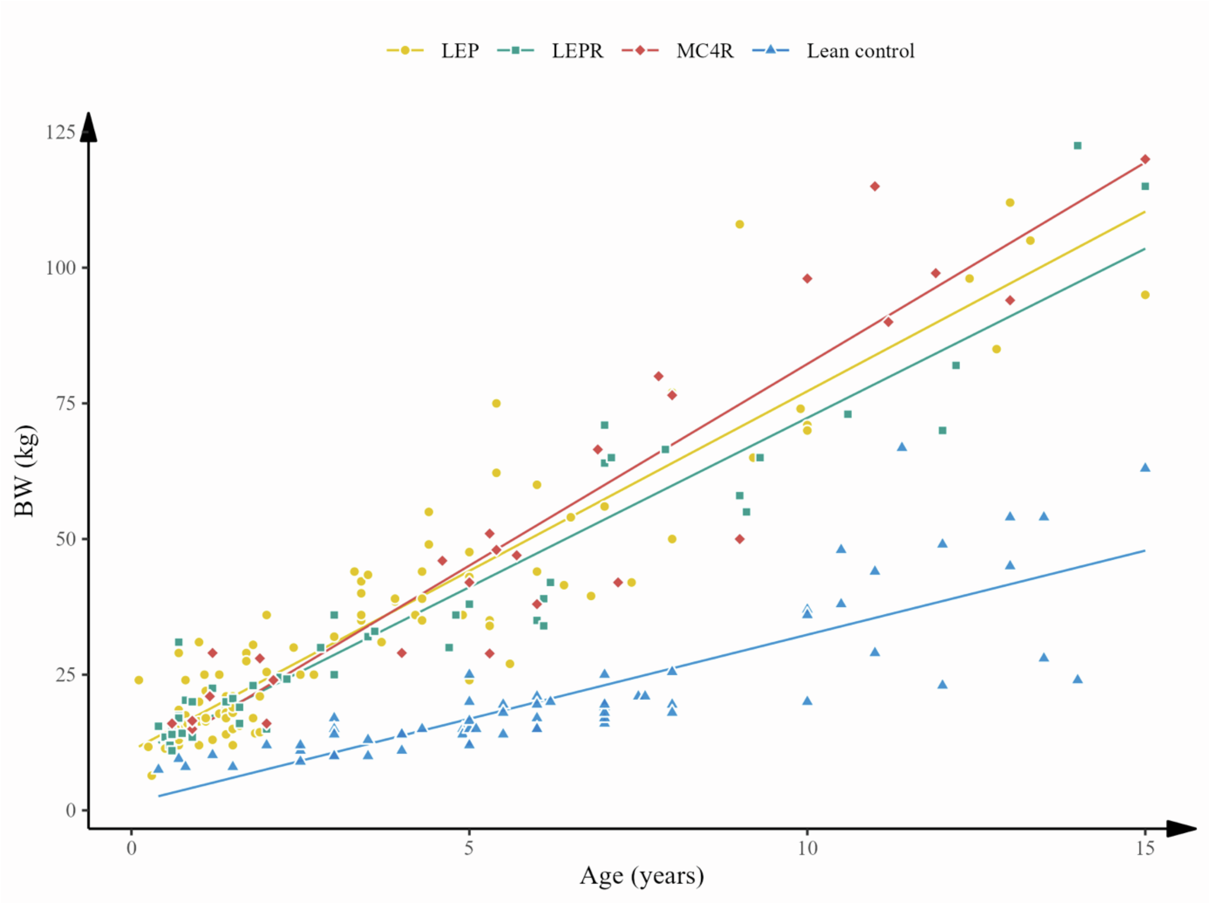

Supplementary Figure 3. Age-related changes in body weight (BW) in *LEP, LEPR* and M*C4R* deficient children and normal body weight controls. Related to Results: Body Growth.
